# Oxalate nephropathy and chronic turmeric supplementation: a case report

**DOI:** 10.1590/2175-8239-JBN-2023-0079en

**Published:** 2024-01-15

**Authors:** Onica Washington, Emily Robinson, Deetu Simh, Hemant Magoo, Ashish Verma, Helmut Rennke, Reza Zonozi

**Affiliations:** 1 Brigham and Women’s Hospital, Division of Nephrology, Boston, Massachusetts, USA.; 2 Saint Vincent Hospital, Division of Nephrology, Worcester, Massachusetts, USA.; 3 Brigham and Women’s Hospital, Department of Pathology, Boston, Massachusetts, USA.; 4 Massachusetts General Hospital, Division of Nephrology, Boston, Massachusetts, USA.

**Keywords:** Oxalate Nephropathy, Turmeric, Curcumin, Nefropatia por Oxalato, Cúrcuma, Curcumina

## Abstract

We present a case of a 69-year-old man who presented for routine check-up and was incidentally found to have kidney failure with an initially unrevealing history and bland urinary sediment. He was diagnosed with oxalate nephropathy in the setting of chronic turmeric supplementation and chronic antibiotic therapy with associated diarrhea. Our case provides several key insights into oxalate nephropathy. First, the diagnosis requires a high index of clinical suspicion. It is uncommonly suspected clinically unless there is an obvious clue in the history such as Roux-en-Y gastric bypass or ethylene glycol poisoning. Diagnosis can be confirmed by histopathologic findings and corroborated by serum levels of oxalate and 24-hour urinary excretion. Second, the diagnosis can often be missed by the pathologist because of the characteristics of the crystals unless the renal pathologist has made it a rule to examine routinely all H&E sections under polarized light. This must be done on H&E, as the other stains dissolve the crystals. Third, one oxalate crystal in a routine needle biopsy is considered pathologic and potentially contributing to the AKI or to the CKD in an important way. Fourth, secondary oxalosis can be largely mitigated or prevented in many cases, especially iatrogenic cases. This can come through the surgeon or the gastroenterologist providing proper instructions to patients on an oxalate-restricted diet or other specific dietary measures. Lastly, this case highlights the success that results from cooperation and communication between the pathologist and the treating physician.

## RESUMO

Relatamos o caso de um homem de 69 anos que se apresentou para exame de rotina e descobriu-se incidentalmente que ele tinha insuficiência renal, com histórico inicialmente não revelador e sedimento urinário brando. Ele foi diagnosticado com nefropatia por oxalato no contexto de suplementação crônica de cúrcuma e antibioticoterapia crônica com diarreia associada. Nosso caso fornece diversas sugestões importantes sobre nefropatia por oxalato. Primeiro, o diagnóstico requer elevado índice de suspeita clínica. A suspeita clínica é incomum, a menos que haja evidência óbvia no histórico, como bypass gástrico em Y de Roux ou envenenamento por etilenoglicol. O diagnóstico pode ser confirmado por achados histopatológicos e corroborado por níveis séricos de oxalato e excreção urinária de 24 horas. Segundo, o diagnóstico pode passar despercebido pelo patologista devido às características dos cristais, a menos que o patologista renal estabeleça como regra examinar rotineiramente todas as seções coradas com H&E sob luz polarizada. Isso deve ser feito com H&E, pois, outras colorações dissolvem os cristais. Em terceiro lugar, um cristal de oxalato em biópsia por agulha de rotina é considerado patológico, contribuindo potencialmente para LRA ou para DRC de maneira significativa. Em quarto lugar, a oxalose secundária pode ser amplamente mitigada ou prevenida em muitos casos, especialmente casos iatrogênicos. Isso pode ser feito pelo cirurgião ou pelo gastroenterologista, fornecendo instruções adequadas aos pacientes sobre uma dieta restrita em oxalato ou outras medidas dietéticas específicas. Por fim, esse caso destaca o sucesso que resulta da cooperação e comunicação entre o patologista e o médico assistente.

## Case Presentation

A 69-year-old Caucasian man presented to his primary care physician (PCP) for a well visit. Laboratory evaluation showed incidental elevation in serum creatinine (SCr) to 3.14 mg/dL (eGFR 19 mL/min/1.73 m^2^ by serum creatinine), with baseline 1.1 mg/dL (eGFR 73 mL/min/1.73 m^2^) six months prior. He felt well, with no complaints other than chronic groin pain. Four years prior to presentation, he developed groin pain that was attributed to prostatitis, for which he received several rounds of fluoroquinolone antibiotic therapy complicated by chronic non-infectious diarrhea. Three years prior, he underwent decompression surgery for pudendal nerve entrapment, for which he received ibuprofen 1200 mg daily for two months for pain control. His review of systems was negative, with no fever, weight loss, respiratory symptoms, musculoskeletal symptoms, or urinary symptoms. He lived at home with his wife in the New England area. There was no recent travel. He denied any known insect bites. There were no high-risk occupational exposures. His past medical history also included dyslipidemia. His family history included a father with nephrolithiasis. His medications included tamsulosin, simvastatin, pregabalin, fluticasone nasal spray, and supplementation with vitamins B6 and B12 and calcium carbonate. There were no recent medication changes. He had no known drug allergies. He denied any smoking or illicit drug use. There was no significant alcohol history. On exam, his blood pressure was 159/72 mm Hg. There was no edema. The remainder of his physical exam was normal. Laboratory testing revealed a hemoglobin of 11.2 g/dL and serum albumin of 4.2 g/dL. Urinalysis showed small leukocyte esterase and no hematuria or proteinuria. The urine sediment was bland. Serologic evaluation for autoimmune disease was negative. The remainder of laboratory results is summarized in Table 1. He was subsequently hospitalized for evaluation and management of kidney failure. A diagnostic procedure was performed.

**Table 1.** Initial or early laboratory evaluation

	Lab value	Reference range
Sodium (mmol/L)	135	137–146
Potassium (mmol/L)	4.2	3.5–5.3
Chloride (mmol/L)	100	98–107
Carbon dioxide (mmol/L)	23	23–32
BUN (mg/dL)	83	5–25
Creatinine (mg/dL)	3.14	0.6–1.4
Calcium (mg/dL)	8.9	8.6–10.3
Albumin (g/dL)	4.2	4.0–5.0
Aspartate aminotransferase (U/L)	15	10–49
Alkaline phosphatase (U/L)	69	35–130
Hemoglobin (g/dL)	11.2	12.0–17.0
Hematocrit (%)	35.8	35.0–50.0
Platelet count (per mm^3^)	159,000	150,000–400,000
**Urinalysis**		
pH	5.0	5.0–8.0
Blood	Negative	Negative
Glucose	Negative	Negative
Ketones	Negative	Negative
Protein	Negative	Negative
Leukocyte Esterase	Small	Negative
Red blood cell (per high powered field)	1	0–2
White blood cell (per high powered field)	4	0–5
Urine Random Total Protein (mg/dL)	<4.0	<4.0

* Serum alanine aminotransferase and urinary albumin/creatinine ratio were not collected at the initial hospital visit.

## Differential Diagnosis

The differential diagnosis was formulated around the following salient features of the case: a 69-year-old man with rapidly worsening kidney function without hematuria or proteinuria in the setting of chronic pudendal nerve pain - initially attributed to prostatitis, then subsequently attributed to pudendal nerve entrapment, chronic antibiotic-associated diarrhea, and chronic exposure to non-steroidal anti-inflammatory drugs (NSAID).

### Obstructive Nephropathy Secondary to Bladder Dysfunction Secondary to Pudendal Nerve Entrapment

The pudendal nerve is a motor and sensory nerve originating from the second, third, and fourth sacral nerve roots. The nerve travels to three areas after leaving the sacral plexus: the gluteal region, the pudendal canal, and the perineum. As the bladder fills with urine, the pudendal nerve contracts the external urethral sphincter which closes the urethra. The nerve is also involved in coordinating relaxation of the urethral sphincters, allowing the bladder to void. Lesions of the pudendal nerve have been associated with voiding dysfunction^1^.

Between 7–24% of the population are diagnosed with “chronic pelvic pain” or pudendal pain, vulvodynia, pudendal neuralgia, or chronic proctalgia^1^. These pain syndromes remain poorly understood, and guidance regarding diagnoses and treatments is still limited. Our patient had a significant history of chronic pelvic pain and underwent pudendal nerve decompression surgery. He had no signs or symptoms of urinary retention prior to or after surgery, as well as no changes to the caliber or force of his urinary stream, no urinary frequency or urgency or irritative bladder symptoms. He had no history of benign prostatic hyperplasia (a common cause of bladder outlet obstruction in older men). Furthermore, he had a kidney ultrasound showing a right and left kidney size of 10.6 cm and 12.2 cm, respectively, without evidence of hydronephrosis.

### Allergic Interstitial Nephritis Secondary to Nsaid Use

Allergic interstitial nephritis is characterized histologically by renal interstitial edema and the presence of inflammatory cells. Inflammatory infiltrates are comprised primarily of lymphocytes, macrophages, eosinophils, and plasma cells. Typically, the glomeruli and vessels are unaffected. Fibrotic changes can be seen within 7–10 days if the inflammatory process continues unabated.

Interstitial nephritis is a frequent cause of acute kidney injury and can be associated with rash, low-grade fevers, and peripheral eosinophilia and eosinophiluria. Drug-induced interstitial nephritis accounts for most cases^2^. Theoretically, any drug can lead to the development of interstitial nephritis, and the list of medications implicated in interstitial nephritis is ever-expanding. Additionally, interstitial nephritis can be associated with infections (e.g., cytomegalovirus, *Streptococcus*) and inflammatory and autoimmune conditions (e.g., sarcoidosis, systemic lupus erythematosus); however, in many cases, interstitial nephritis is idiopathic.

The mainstay of treatment continues to be the withdrawal of the offending agent and the administration of glucocorticoids. However, glucocorticoids have no definitive data suggesting that they are beneficial in the case of NSAID-induced interstitial nephritis, which can be associated with nephrotic syndrome and T-cell infiltration^3^. There have been documented reports that interstitial nephritis resistant to steroids may benefit from treatment with immunosuppressive regimens such as cyclophosphamide, cyclosporine, or mycophenolate mofetil^4^.

### Pre-Renal Azotemia Secondary to Volume Depletion from Diarrhea

The possibility of this being a case of pre-renal azotemia is supported by the combination of a supportive history of GI fluid losses and a bland urinary sediment. Although the patient did not show overt evidence of volume depletion on clinical exam, assessment of fluid responsiveness by giving IV fluids then monitoring the trajectory of serum creatinine could be a helpful diagnostic and therapeutic test.

## Histopathology

**Figure 1.**
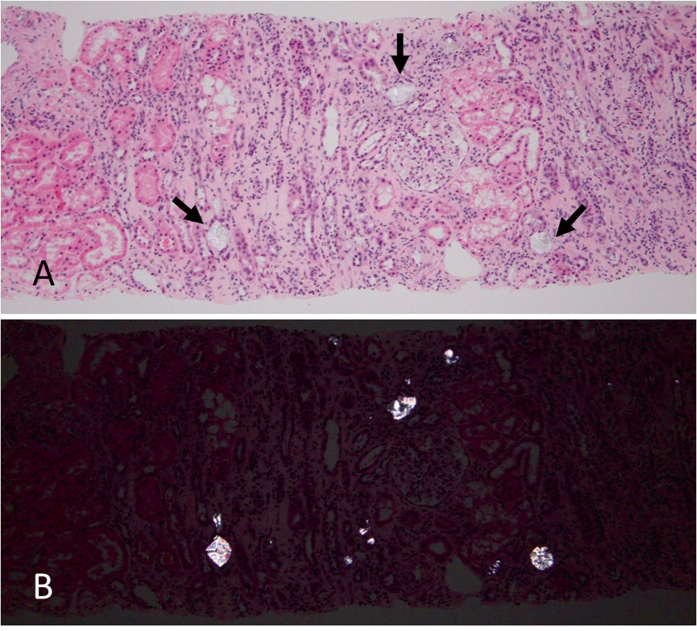
Light microscopy of patient’s kidney biopsy. A. Paraffin section stained with hematoxylin and eosin (H&E) showing mild interstitial nephritis and acute tubular injury highlighted by the distention of the tubules. The arrows point to crystals of calcium oxalate. B. Same histologic section viewed under polarized light revealing birefringence (dichroism) of the calcium oxalate crystals.

**Figure 2.**
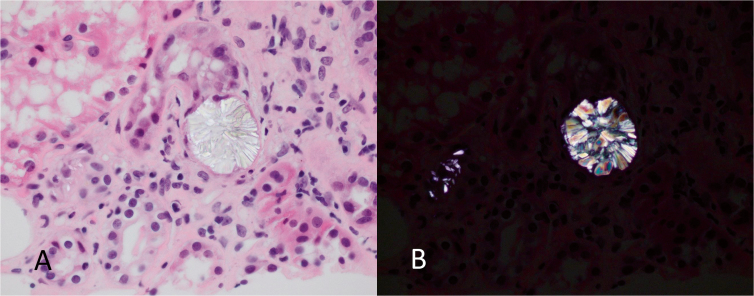
A. Calcium oxalate crystal within a distended tubule at higher magnification (H&E). B. The crystals show a characteristic iridescence when viewed under polarized light.

The kidney biopsy was evaluated by routine light, immunofluorescence, and electron microscopy. The sample consisted of cortex and medulla and it included 48 glomeruli, 3 of which showed global sclerosis. The cortex revealed moderate tubular atrophy, interstitial fibrosis, and mild infiltration by mononuclear inflammatory cells (Figure 1A). Several tubules contained crystals that were revealed more easily under polarized light (Figure 1B). The crystals were colorless, refractile, irregularly shaped, and showed a characteristic iridescence (Figure 2A and B). These crystals are only preserved in the sections stained with hematoxylin and eosin (H&E), as they are solubilized in the steps used in most other special stains. The immunofluorescence microscopy revealed only scattered fibrin in the interstitium and in the lumen of some tubules. Deposits of immunoglobulin or complement were not seen in the glomeruli or along the tubular basement membranes (not shown). Electron microscopy showed mild expansion of the mesangial matrix, minimal thickening of the glomerular basement membranes, and mild nonspecific changes in the tubules not affected by the calcium oxalate crystals.

## Final Diagnosis

The final diagnosis was: widespread calcium oxalate deposits in the tubules (renal oxalosis) associated with acute tubular injury, chronic interstitial nephritis, and extensive tubular atrophy and interstitial fibrosis.

### Follow-Up

On initial admission to the hospital, the patient received glucocorticoids for presumed interstitial nephritis. Unfortunately, his kidney failure continued to worsen and he was initiated on kidney replacement therapy. A kidney biopsy was performed. After the biopsy revealed renal oxalosis, the pathologist contacted the treating nephrologist to discuss the findings and review possible causes of primary and secondary oxalosis in this patient. The nephrologist interviewed the patient again in search of an identifiable cause for the oxalosis. Upon further questioning, the patient reported he has been taking 2 grams of turmeric (curcumin) daily for the last two years, with the aim of it serving as an anti-inflammatory to alleviate chronic pain from nerve entrapment. Further testing revealed a serum oxalate level (obtained after starting dialysis) of 14.4 µmol/L (reference range < 1.9 µmol/L). His 24-hour urinary oxalate level was 68 mg (reference range 7–24 mg). Additionally, he was noted to have a 24-hour urine citrate of 40 mg (reference range > 450 mg/day). Genetic testing was negative for any pathogenic variants in the genes coding for alanine-glyoxylate aminotransferase, D-glycerate dehydrogenase, or 4-hydroxy-2 oxoglutarate aldolase. The pathologist was informed of the turmeric exposure after contacting the nephrologist again prior to finalizing the pathology report. The turmeric supplementation was discontinued, and the patient was counseled to avoid NSAIDs. He began an oxalate-restricted diet, which involved avoiding spinach, chocolate, and black tea. He started potassium citrate 20 mEq daily to address the hypocitraturia. He also started calcium carbonate supplementation with meals to bind to dietary oxalate. Four months after starting therapy, his urine output increased. A 24-hour urine creatinine clearance was 29 mL/min. Hemodialysis was discontinued. One year after discontinuing dialysis, he remains well with stable CKD with an eGFR of 22 mL/min/1.73 m^2^. He is currently listed for kidney transplantation.

## Discussion

This case report of a patient with severe kidney injury with an initially unrevealing history and bland urinary sediment provides several key insights into oxalate nephropathy. First, the diagnosis requires a high index of clinical suspicion. Oxalate nephropathy is uncommonly suspected clinically unless there is an obvious clue in the history such as Roux-en-Y gastric bypass or ethylene glycol poisoning (a minority of cases). This presumptive diagnosis should be routinely considered for any case of acute kidney injury (AKI) or AKI on chronic kidney disease (CKD) of unclear etiology. Diagnosis can be confirmed by histopathologic findings and corroborated by serum levels of oxalate and 24-hour urinary excretion. Second, the diagnosis can often be missed by the pathologist because of the characteristics of the crystals, unless the renal pathologist has made it a rule to routinely examine all H&E sections under polarized light. This must be done with H&E, as the other stains dissolve the crystals. Third, a single oxalate crystal in a routine needle biopsy is considered pathologic and potentially contributing to AKI or CKD in an important way. Fourth, secondary oxalosis can be largely mitigated or prevented in many cases, especially those cases that are iatrogenic. This can come through the surgeon or the gastroenterologist providing proper instructions to patients on an oxalate-restricted diet or other specific dietary measures as outlined below. Lastly, this case highlights the success that results from cooperation and communication between a pathologist and clinician.

Oxalate is the anion of oxalate acid, which is derived both exogenously from diet and endogenously from normal metabolism. It is primarily excreted in the urine, mostly through glomerular filtration, but also with tubular secretion via the SLC26 transporter family^5^. When serum levels accumulate, it results in hyperoxaluria.

This patient had chronically taken high doses of turmeric supplementation. Turmeric, with the active ingredient curcumin, has a relatively high content of oxalate, estimated at 1969 mg oxalate per 100 grams of turmeric^6^. Our patient consumed 2 grams of turmeric daily, which corresponds to approximately 40 mg of oxalate daily. Furthermore, his chronic antibiotic use may have led to altered colonic flora, which can disturb floral bacterial oxalate metabolism. These can further lead to high serum oxalate concentrations. As the serum oxalate levels rise, it can deposit in the kidney, resulting in an inflammatory response, namely tubulointerstitial nephritis. And lastly, the cause of his hypocitraturia was unverified, but one possibility is the hypocitraturia was secondary to gastrointestinal bicarbonate wasting from his chronic diarrhea, resulting in acidosis, which decreases renal citrate excretion^7^. The absence of a metabolic acidosis on laboratory testing, however, may provide an argument against this.

The kidney biopsy demonstrated features consistent with a diagnosis of oxalate nephropathy (also called renal oxalosis) and acute interstitial nephritis and tubular injury. Oxalate nephropathy is a histopathologic diagnosis characterized by tubular deposition of calcium oxalate crystals, causing an inflammatory response resulting in interstitial nephritis and eventual interstitial fibrosis and tubular atrophy. Diagnosis depends on assessing the tissue under polarized light. In a review by Rosenstock et al.^8^, the reported prevalence was as high as 4.07% in a biopsy cohort from the New York City metropolitan area, but further epidemiological investigation is needed.

Oxalate nephropathy can result from primary and secondary hyperoxaluria (Table 2). Primary hyperoxaluria (PH) is a group of inborn metabolic errors leading to overproduction of oxalate by the liver because of the liver’s inability to process oxalate’s precursor, glyoxylate. Glyoxylate is converted to oxalate with lactate dehydrogenase. Oxalate is poorly soluble and binds to calcium. The higher filtration of oxalate in the kidneys results in more oxalate deposition in the kidneys, which can manifest as nephrocalcinosis, kidney stones, and end-stage kidney disease. Indeed, it accounts for 1-2% of cases of pediatric end-stage kidney disease^8^. There are three types of PH based on the enzymatic defect, with type 1 (defect in alanine glyoxylate aminotransferase) being the most clinically severe. A diagnosis of PH is based on the combination of clinical features of recurrent calcium stones, oxalate crystals in the urine sediment, a biopsy showing oxalate deposition or imaging showing nephrocalcinosis, an elevated urinary oxalate level, and confirmatory genetic testing for pathogenic variants in the associated genes (*AGXT*, *GrHPR*, and *HOGA1*). In cases of reduced kidney function, urinary oxalate levels may be reduced, and therefore an elevated plasma level can be used. In cases of negative genetic testing, a liver biopsy can be performed with staining for the hepatic enzyme AGT, the absence of which is confirmatory.

**Table 2.** Causes of oxalosis

Primary hyperoxaluria	Inborn errors of metabolism Type 1: Alanine-glyoxylate aminotransferase deficiency Type 2: D-glycerate dehydrogenase deficiency Type 3: 4-hydroxy-2oxoglutarate aldolase
Secondary (enteric) hyperoxaluria 4 categories of secondary causes 1. Fat malabsorption 2. Increased dietary intake 3. Increased dietary intake of precursors 4. Disturbance of intestinal flora	Fat malabsorption Roux-en-Y gastric bypass Partial gastrectomy Exocrine pancreatic insufficiency Inflammatory bowel disease Use of orlistat (lipase inhibitor, used as weight loss drug) Short bowel syndrome
	Increased dietary oxalate Spinach, rhubarb, chocolate, pepper, black tea, soy products, beans, potatoes, turmeric, nuts
	Increased dietary precursors of oxalate Mega-doses of vitamin C Ethylene glycol poisoning
	Disturbance of intestinal flora Through elimination of normal colonic *Oxalobacter formigenes* through antibiotic use Antibiotics associated with risk of stone formation Fluoroquinolones Cephalosporins Nitrofurantoin Broad spectrum penicillin (Amoxicillin, Penicillin G) Sulfa-containing antibiotics (Sulfamethoxazole-trimethoprim)

In contrast to PH, secondary (enteric) hyperoxaluria is due to an acquired increased intestinal absorption of oxalate. This could be due to fat malabsorption, increased dietary oxalate, increased dietary precursors of oxalate, or disturbances of intestinal flora. Regarding fat malabsorption, normally ingested oxalate binds to intestinal calcium to form insoluble calcium oxalate, which is excreted in the feces. However, colonic absorption of soluble oxalate is increased if calcium becomes unavailable, which is the case when free calcium binds to free fatty acids during fat malabsorption. This phenomenon is seen after Roux-en-Y gastric bypass or partial gastrectomy, as well as in exocrine pancreatic insufficiency or inflammatory bowel disease^5,8^.

Regarding high dietary oxalate ingestion, there are certain foods rich in oxalate, which includes rhubarb, spinach, beetroot, kiwi, chocolate, tea, cinnamon, and turmeric^9^. The amount absorbed, however, varies by food item^6^. In a study by Tang et al.^6^, subjects were given 3.0 grams of cinnamon daily or 2.8 grams of turmeric daily for 4 weeks (which provided approximately 55 mg of oxalate per day). They found the percentage of oxalate that was water soluble differed markedly between cinnamon (6%) and turmeric (91%). Moreover, only turmeric and not cinnamon led to a significantly increased urinary oxalate level, which highlights the differential absorption of oxalate.

Another means to higher systemic oxalate is through ingestion of oxalate precursors. Two notable examples are ascorbic acid (vitamin C) and ethylene glycol. The intake of ascorbic acid in excess quantities has been associated with calcium oxalate nephrolithiasis. Consumption of ethylene glycol can also result in calcium oxalate deposition and kidney failure^5^.

Lastly, *Oxalobacter formigenes* is an aerobic Gram-negative bacterium that normally colonizes the human colon. It metabolizes oxalate into formic acid and carbon dioxide, thereby lowering colonic absorption of oxalate^5^. Chronic antibiotic therapy is thought to deplete intestinal *Oxalobacter*, leading to elevated levels of oxalate and consequently an increased risk for hyperoxaluria. Data from recent studies suggest an association between antibiotic use and nephrolithiasis^5,10^.

Notably, in contrast to the risks to the kidneys posed by the oxalate content in turmeric supplements, cellular and animal studies suggest that curcumin itself may be beneficial to the kidney. Specifically, curcumin may help restore tubular epithelial cell function through reducing expression of inflammatory cytokines, scavenging reactive oxygen species, limiting apoptosis, and improving mitochondrial homeostasis^11^. Randomized clinical trials in humans are needed to better understand the clinical benefits and risks of turmeric supplementation.

Treatment of oxalate nephropathy depends on the type of hyperoxaluria. General measures for both types include vigorous fluid intake to reduce concentrations of urinary calcium and oxalate to prevent their precipitation, and urine alkalinization with potassium citrate to reduce urinary calcium oxalate saturation. The goal is to maintain urine pH between 6.2 and 6.8^12^. For primary hyperoxaluria, pyridoxine (vitamin B6) supplementation is helpful in PH type 1 because pyridoxine acts as a cofactor for the enzyme AGT, and high doses can stabilize the enzyme. Once the patient develops advanced CKD (eGFR < 30 mL/min/1.73 m^2^), transplantation preparations should be made. The enzymatic defect in PH1 is liver-specific, therefore the curative treatment is pre-emptive liver transplantation or, if indicated, simultaneous or sequential liver-kidney transplantation. Recently, lumasaran has become the first-line of treatment for PH type 1. It is a synthetic double-stranded interfering RNA molecule that inhibits the hydroxyacid oxidase 1 (HAO1) messenger RNA in hepatocytes. This gene normally encodes glycolate oxidase. When glycolate oxidase activity is reduced, there is less glyoxylate, and therefore less oxalate production^13^. For secondary hyperoxaluria, dietary oxalate restriction is paramount. Also, concurrent ingestion of calcium (or dairy products) with foods high in oxalate can lower systemic absorption by binding to oxalate and resulting in fecal excretion. Supplementation with probiotics containing *O. formigenes* appears to be an attractive therapy for hyperoxaluria, but has not been validated as effective therapy in human trials^8^. More recently, the drug reloxaliase (a recombinant oxalate decarboxylase) has been shown to lower urine and plasma oxalate levels in those with CKD. Reloxaliase is now under study in a randomized trial^8^.

In summary, our case reminds us of the importance of having a high index of suspicion for oxalate nephropathy, the importance of communication with the pathologist in uncovering a diagnosis, the potentially severe risks of routine over-the-counter supplements, and the need for further human trials on turmeric supplementation to assess clinical risks versus benefits.
